# Heterotrophic Protists in Hypersaline Microbial Mats and Deep Hypersaline Basin Water Columns

**DOI:** 10.3390/life3020346

**Published:** 2013-05-22

**Authors:** Virginia P. Edgcomb, Joan M. Bernhard

**Affiliations:** Geology and Geophysics Department, Woods Hole Oceanographic Institution, Woods Hole, MA 02543, USA; E-Mail: jbernhard@whoi.edu

**Keywords:** hypersaline, protist, eukaryote, microbialite, saltern, DHAB, Hamelin Pool, E. Mediterranean Sea, foraminifera, SSU rRNA

## Abstract

Although hypersaline environments pose challenges to life because of the low water content (water activity), many such habitats appear to support eukaryotic microbes. This contribution presents brief reviews of our current knowledge on eukaryotes of water-column haloclines and brines from Deep Hypersaline Anoxic Basins (DHABs) of the Eastern Mediterranean, as well as shallow-water hypersaline microbial mats in solar salterns of Guerrero Negro, Mexico and benthic microbialite communities from Hamelin Pool, Shark Bay, Western Australia. New data on eukaryotic diversity from Shark Bay microbialites indicates eukaryotes are more diverse than previously reported. Although this comparison shows that eukaryotic communities in hypersaline habitats with varying physicochemical characteristics are unique, several groups are commonly found, including diverse alveolates, strameonopiles, and fungi, as well as radiolaria. Many eukaryote sequences (SSU) in both regions also have no close homologues in public databases, suggesting that these environments host unique microbial eukaryote assemblages with the potential to enhance our understanding of the capacity of eukaryotes to adapt to hypersaline conditions.

## 1. Introduction

Hypersaline waters (generally >10% NaCl; [1]) have salinities that exceed the 3.5% total salt of most oceans. These include salt or soda lakes, salterns, coastal lagoons, and deep hypersaline anoxic brines, and contain Bacteria, Archaea and Eukarya [2]. Hypersaline environments are characterized by a low water content or water activity (a_w_) because of the high-salt concentrations [1]; this presents challenges for organisms living in these habitats. With increasing salinity, halophilic (requiring salinities greater than normal seawater salinity) and halotolerant (able to survive salinities greater than normal seawater) eukaryotes appear to comprise smaller fractions of communities. Typically, growth does not occur at a_w_ below 0.72 because, below this, there is not enough water available for dissolving nutrients, general metabolic processes, and for hydrating proteins and nucleic acids [3]. Other stresses may also be present in hypersaline habitats, such as intense solar radiation and elevated temperatures in shallow lakes, solar salterns and salt production plants, intense pressures in deep hypersaline anoxic basins, and vastly different ionic compositions of the salts themselves. For instance, if the salt in the brine originates from seawater (thalassohaline) it will be dominated by sodium chloride, however, if the salts originate from other sources (athalassohaline), other ions will dominate, and the ionic composition can vary widely [1].

To prevent loss of cellular water to the environment, microbial eukaryotes (and other halotolerant and halophilic organisms) require a way to balance the osmotic pressure created by their hypersaline habitat. Some halophiles accumulate solutes within the cytoplasm and others use sodium pumps to expel sodium ions out of the cell while concentrating potassium ions inside the cell to balance osmotic pressure [4]. Some halotolerant algae are known to balance osmotic pressure by producing or taking up organic molecules from the environment, such as glycerol [1]. A full discussion of adaptive mechanisms of microorganisms to hypersaline habitats is outside the scope of this review. Extensive literature exists describing the adaptive mechanisms for Bacteria and Archaea and, to a lesser extent, Eukarya to hypersaline conditions, including high guanine to cytosine ratios in DNA, high concentrations of acidic residues on exteriors of proteins, and unique lipids, cellular architectures, pigments, physiologies and metabolisms (e.g., [1]; and extensively discussed in [5]). 

Protists are an essential component of microbial food webs that play a central role in global biogeochemical cycles, thus making them key players in sustaining the healthy functioning of any ecosystem. Protists include autotrophic (capable of making organic molecules from inorganic sources via photosynthesis, e.g., algae) and heterotrophic (those that prey on preformed organic carbon, including other microbes, and hence contribute to pools of dissolved organic carbon through release of metabolic wastes and “sloppy feeding”, e.g., ciliates and flagellates) microbial eukaryotes. Microscopical observations of hypersaline habitats long ago revealed heterotrophic protists to be present (e.g., [6,7,8,9,10] and more recently, [11,12,13]). However, studies indicated protists were rare or nonexistent in extremely hypersaline (over 30%) environments (e.g., [13,14,15,16,17,18]) (for contrasting viewpoint see [19]). Autotrophs and heterotrophs of moderately hypersaline habitats (6–15%) are suspected of being euryhaline representatives of marine forms that have adapted to life in extremely salty conditions (e.g., [10,11,12,13,20,21]. Such studies based primarily upon microscopic observations can sometimes underestimate diversity, particularly with respect to heterotrophic nanoflagellates and naked amoebae (discussed in [22]). The heterotrophic nanoflagellates, ciliates and amoebae appear to be the major groups of protists adapted to life in hypersaline environments (e.g., [12]). Most investigations of halophilic ciliate and flagellated protists have examined habitats with 10–20% salinities, and some of the taxa identified appear ubiquitous in hypersaline environments (e.g., the marine green flagellate *Dunaliella* spp. in hypersaline lakes; [16]), while some appear uniquely adapted to specific ratios of particular ions (e.g., [23]). The physiological underpinnings of this remain to be determined.

Heterotrophic bicosoecids and non pigmented chrysomonads belonging to stramenopiles are protists known to be important components of many aquatic microbial communities [24]. Halotolerant and halophilic bicosoecids have been isolated from hypersaline locations that can grow in both normal seawater and 17.5% salinity; these have not been previously reported in marine or freshwater environments using culturing or PCR-based approaches [22]. At higher salinities (up to 18%), the diversity of stramenopiles appears to be significantly less than in typical marine conditions, consisting primarily of *Halocafeteria*, and other “Cafeteria” species [22]. Investigations of higher salinities (e.g., deep brine basins with salinity of 28%) did not reveal stramenopiles [25]. Dinoflagellates are another group of heterotrophic protists frequently documented in environments with salinities between 6 and 30% (e.g., [11,26,27,28]).

Our view of heterotrophic protist diversity in hypersaline environments is expanding, particularly with additional data from molecular-based surveys of these habitats. There is increasing evidence of extremely halophilic heterotrophs that are distinct from marine or freshwater forms, such as the ciliate *Trimyema koreanum* sp. nov. in solar salterns with salinity of 29% [29], and a diversity of other heterotrophs from nearly saturated brines (30% or more salinity) that could not grow at salinities less than 7.5% [29,30,31,32]. Additionally, there are molecular and microscopic observations of heterotrophic protists in habitats up to 36% salinity (e.g., [25,33,34]). This paper compares heterotrophic protists in two shallow hypersaline habitats: hypersaline microbial mats in solar salterns and microbialites in Shark Bay, Australia, with deep anoxic brines at the bottom of the Mediterranean Sea. We focus on heterotrophs because autotrophic communities in deep-sea hypersaline habitats are not comparable to shallow water communities within the photic zone. There are many physicochemical differences between these habitats that likely contribute to variations in microbial communities between locations, including water depth, temperature, salinity, salt ionic composition, concentrations of sulfide and methane, *etc*. A comparison of protist populations in these habitats reveals that protists are present in hypersaline waters and sediments, and that physicochemical differences in habitats select for unique protist populations in different hypersaline habitats.

## 2. Views of Eukaryotic Diversity in Selected Hypersaline Habitats

### 2.1. Microbialites in Hypersaline Shark Bay, Australia

**Figure 1:**
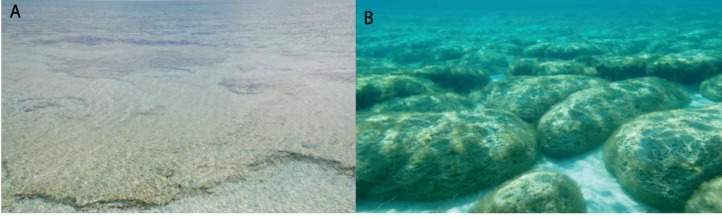
Hamelin Pool, Shark Bay, W. Australia microbialites. (**A**) smooth mat; (**B**) colloform mat.

Hamelin Pool, in Shark Bay, Western Australia, is one of the few sites where microbialites, or lithifying microbial mats, are forming today (Figure 1). These microbialites, are found along the margins of Hamelin Pool [35], and form by trapping and binding of carbonates by extracellular polymeric substances (EPS), produced by filamentous cyanobacteria and other bacteria (e.g., [36]). Because fossilized microbialites comprise the earliest visible record of life on Earth (e.g., [37,38]), the modern Hamelin Pool microbialites have been intensively studied. Waters of Hamelin Pool are typically 6–7% salinity.

Tong [39] reported 41 different species of heterotrophic nanoflagellates based on light microscopic observations of hypersaline waters of Shark Bay. These included apusomonads, cercomonads, choanoflagellates, cryptomonads, euglenids, heteroloboseids, stramenopiles, and several groups of uncertain taxonomic affiliation. When sediments from four different sites in the Western Australia Shark Bay area with differing salinities were examined, fewer species were observed to overlap with previously documented species from normal marine environments in the most hypersaline samples (four times the salinity of seawater) than samples from waters that were only two times the salinity of seawater [11]. This finding is consistent with the generally accepted idea that eukaryotic microbial diversity declines as hypersalinity increases, as discussed above.

Examination of eukaryotic microbial diversity has been more limited than investigations of prokaryotic diversity in Shark Bay microbialites. Al-Qassab *et al*. [21] identified flagellate protists in modern stromatolites in Shark Bay, and foraminiferal tests (shells) were observed in some thrombolites [40]. Different microbialite types have varied degrees of lamination, and groups of eukaryotes such as the foraminifera, which are known bioturbators of sediments, have the potential to influence sediment fabric (discussed in Bernhard *et al*., 2013 [41]).

Using a combination of next generation and Sanger-based sequencing approaches we gathered as comprehensive a picture of eukaryotic diversity as possible in the 0–1 cm and 1–2 cm intervals of different microbialite samples collected in Hamelin Pool, Shark Bay in June 2011. Salinity is reported in Practical Salinity Units (PSU), which is a measure of salt content of water based upon electrical conductivity of a sample relative to reference standard of seawater. Normal seawater at 15 °C has a salinity of 3.5%, or 35 PSU. The salinity of the overlying water at the Shark Bay sampling site was 66–72 PSU (Table 1). We sampled pustular mats, which are irregular, clotted mats; colloform mats, which are coarse, laminoid wavy mats; and smooth mats, which are fine, laminoid structures (nomenclature according to [35,42]). Total RNA was extracted using the FastRNA Pro Soil-Direct Kit according to the manufacturer’s instructions, with the exception that a Turbo DNase step (Ambion) was included prior to the RNA Matrix cleanup. Total RNA was converted to cDNA and used as a template for all PCR reactions using a Superscript One-Step RT-PCR kit (Invitrogen) and eukaryotic small subunit ribosomal RNA V4 hypervariable region PCR primers [43] or general primers for foraminifera (S14F1/S17, [44]) to focus on the more active fraction of the community. Foraminifera-specific amplifications were necessary because general V4 primers do not detect most foraminifera. Foraminiferal PCR products were cloned into pCR4-TOPO using the TOPO TA Cloning Kit (Invitrogen) for Sanger sequencing (one 96-well plate per microbialite sample), and pyrotags were submitted for Titanium pyrosequencing. Sequence data were processed for quality control, clustered into operational taxonomic units (OTUs) at 97% sequence identity in QIIME [45], and taxonomic assignments were made using JAguc [46].

**Table 1:** Physicochemical data for several Eastern Mediterranean deep hypersaline anoxic basins and Hamelin Pool, Shark Bay. In this context, “interface” equates to the halocline.

Sample	Coordinates		Water Depth (m)	Total Salinity (PSU)	Oxygen (mL/L)
Discovery Interface ^2^	35°19'N	21°41'E	3,580	70 ^1^	0.50
Thetis Interface ^3^	34°40'N	22°08'E	3,259	80	0.68
Thetis Brine ^3^	34°40'N	22°08'E	3,415	340	0
Bannock Brine ^2^	34°17'N	20°00'E	3,790	280	0
Bannock Interface ^2^	34°17'N	20°00'E	3,300	246	0.50
Atalante Upper Interface ^5^	35°18’N	21°23'E	3,499	39	0.44
Atalante Lower Interface ^5^	35°18'N	21°23'E	3,501	365	0
Urania Interface ^6^	35°13'N	21°28'E	3,467	63	1.22
Hamelin Pool microbialites ^7^	26°15'S	114°14'E	0–3	66–72	supersaturated at 0–1 cm, 0 at 1–2 cm
Guerrero Negro saltern mats ^4^	27°41'N	113°55'W	1–2	90	na

^1^ Using the conventional sensor mounted on the Niskin rosette, the measurement of conductivity is not reliable in athalassohaline brines enriched by divalent cations. na = not available. ^2^ Edgcomb *et al*.; 2009 [47]; ^3^ Stock *et al*. 2011 [48]; ^4^ Bebout *et al*. 2002 [49]; ^5^ Alexander *et al*. 2009 [25]; ^6^ Orsi *et al*. 2012 [50]; ^7^ this study.

**Figure 2:**
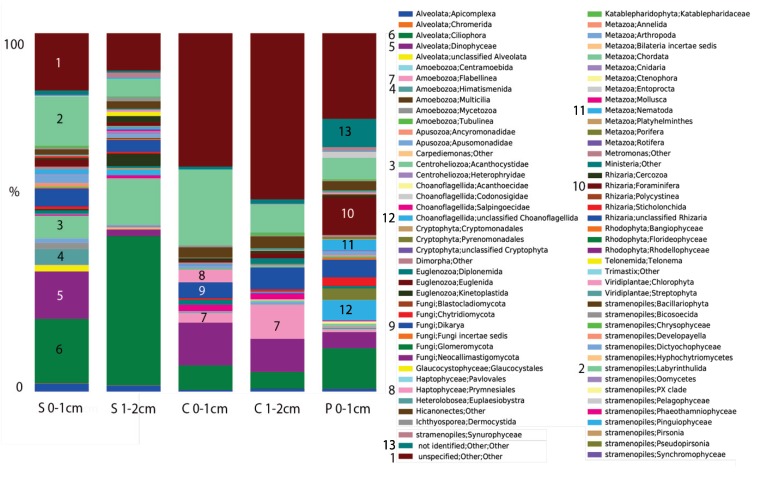
Stacked histogram of eukaryotic operational taxonomic units (out) composition of (97% sequence similarity, weighted data presentation) in Hamelin Pool, Australia microbialite and water samples based on eukaryote sequences (SSU) rRNA signatures (cDNA template). Y-axis corresponds to fraction of OTUs affiliating with each grouping out of 100%. S = smooth mat, C = colloform, P = Pustular.

In contrast to previous analyses on microbial eukaryotes in Shark Bay microbialites, we found diverse communities of eukaryotes in all microbialite samples examined (Figure 2). Eukaryotic signatures were dominated by Alveolata (10–50% of OTUs), unclassified eukaryotes (5–45%), and stramenopiles (10–30%). The dominant alveolates differed in different microbialite types. In colloform mats, alveolates were dominated by Heterocapsaceae and *Protodinium* (Dinophyceae), and more diversity was observed in the 0–1 cm depth interval than in the 1–2 cm depth. In pustular mats, the greatest diversity of alveolates was observed, with OTUs from both ciliates and dinoflagellates. Alveolates in smooth mats were dominated by dinoflagellate OTUs affiliating with Gymnodiniales (40%) in the 0–1 cm interval, and in the 1–2 cm interval, 90% of OTUs affiliated with ciliates (Litostomatea). Stramenopile OTUs were dominated in all mat samples by representatives of labyrinthulids affiliating with Thraustrochytridae (60–85% of stramenopile OTUs). The high number of OTUs that could not be assigned a taxonomy based on BLASTn (<80% sequence similarity to GenBank sequences) suggests the presence of novel eukaryotic lineages in Hamelin Pool microbialites.

The highest number of rhizarian OTUs in the surface 0–1 cm samples was recovered from pustular mat samples. In all other mat samples, differences were observed in foraminiferal OTUs in different microbialite types, and OTUs were detected from within Rotaliida, Textulariida, Milliolina, and Allogromiida (thecate, non-mineralized forms). The latter group is of particular interest because they are modern representatives of basal foraminifera that likely evolved in the Precambrian (e.g., [51]), when microbialites dominated Earth’s biosphere. 

**Figure 3:**
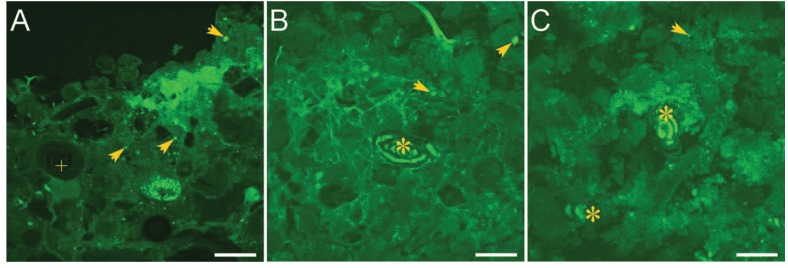
LSCM images of microbialites in FLEC sections from Hamelin Pool. (**A**) Sediment-water interface showing masses of cyanobacteria comprising pustular mat; (**B**) Foraminifer (*) inhabiting smooth mat; (**C**) Foraminifera (*) inhabiting colloform mat. Arrows = indeterminate protists; * = foraminifer; + = ooid with concentric layering. Scales = 200 µm.

As an independent means to assess eukaryotic presence in microbialites, we performed microscopic studies using a viability indicator. CellTracker^®^ Green CMFDA (CTG; Invitrogen) was used as an indicator of active hydrolytic esterase activity in marine populations. The Fluorescently Labeled Embedded Core (FLEC) method [52], which preserves the life positions of microbes in a sample, was also used to assess the living positions of eukaryotes within microbialites by collecting syringe cores of 1.5-cm inner diameter from the same microbialites sampled for sequence analysis. At least five replicate cores were collected per microbialite structure. Along with overlying seawater, cores were incubated with 1 µM CTG at ambient light and temperature for approximately 6–8 hours, after which 3% glutaraldehyde in 0.1 M cacodylate sodium salt buffer was introduced into each core for approximately one hour. Cores were then rinsed three times in buffer and transported to the laboratory for further processing. Processing steps through polymerization followed Bernhard *et al.* [52] except that cores were embedded in LR White rather than the typical Spurrs’ resin. Polymerized cores were sectioned coronally with an Isomet low speed rock saw. Both sides of each section were scanned with a Leica FLIII stereomicroscope equipped with epifluorescence capabilities to identify fluorescent objects suggestive of eukaryotes (size, shape). Promising targets were imaged with an Olympus Fluoview 300 Laser Scanning Confocal Microscope (LSCM). FLEC analysis supports sequence analysis indicating that microbial eukaryotes inhabit the microbialites (Figure 3). For example, foraminifera are easily identified in many FLEC sections (e.g., Figure 3B,C). Other picoeukaryotes (0.2–2 µm) are more difficult to identify, but putative protists can be quite common (Figure 3).

### 2.2. Microbial Mats in Hypersaline Solar Salterns

Extensive microbial mats grow within the hypersaline lagoons of the Exportadora de Sal SA saltern in Guerrero Negro, Baja California Sur, Mexico. Salterns, or salt works, involve a series of sun-baked lagoons where seawater is gradually evaporated, to the point where salts including sodium chloride precipitate, and are harvested. Feazal *et al*. [28] examined eukaryotic diversity in Guerrero Negro mat samples collected from a pond with 90 PSU salinity [44]. The mat cores from which they extracted DNA were 6 cm thick, and were sectioned into 1 mm intervals for 0–6 mm and ~1 cm intervals downcore. The extent of eukaryotic diversity these authors detected using Sanger sequencing of small subunit ribosomal RNA genes (SSU rRNA) was extremely low, with only 15 ribotypes identified among 890 clones analyzed [28]. There are several possible explanations for the low diversity relative to the microbialite samples, including potential primer biases, swamping of clone libraries by metazoan (377 nematode signatures from two taxa out of 890 clones analyzed) SSU rRNA signatures in their DNA-based analysis, and screening clones based on Terminal Restriction Fragment Length Polymorphism (T-RFLP), which may have underestimated diversity. An RNA-based analysis of several of these salterns coupling next generation molecular methods with microscopy would provide further insights into eukaryotic diversity in this hypersaline habitat.

### 2.3. Deep Hypersaline Anoxic Basins in the Eastern Mediterranean Sea (DHABs)

Deep brines on the seafloor exist in different locations, including Orca Basin in the Gulf of Mexico, the Red Sea, and the Eastern Mediterranean Sea. DHAB, which are found in many locations in the Eastern Mediterranean Sea are discussed here, and are thought to have formed through the dissolution of buried Messinian evaporitic deposits, followed by accumulation of brines in sea floor depressions ([53] and references therein).

Several studies of E. Mediterranean DHAB water columns have extended our knowledge of the environmental factors that define the limits of life for microbial eukaryotes and have provided insights into novel eukaryotic diversity in these planktic habitats (e.g., [25,33,42,43,54,55]). Recovery of sequences of many taxonomic groups in these studies with no known homologues in public databases suggests these pelagic habitats harbor organisms with possibly novel metabolic/physiological characteristics.

**Figure 4:**
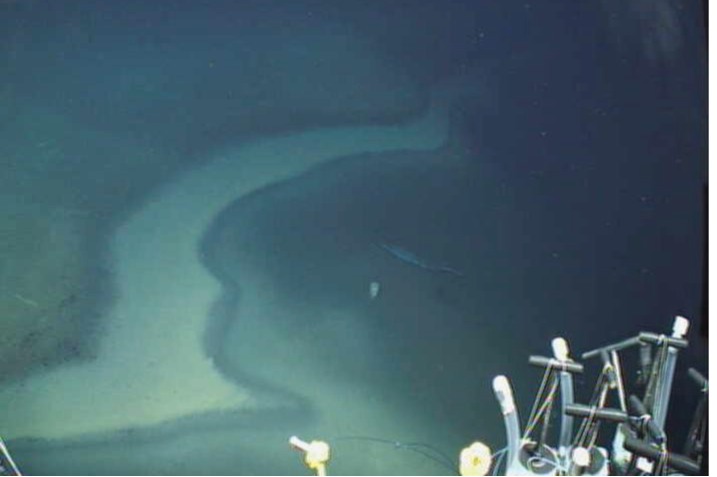
Discovery Basin, Eastern Mediterranean Sea (3,582 m depth). Image taken with ROV *Jason*, showing the Deep Hypersaline Anoxic Basins (DHAB) “beach” (white zone where the halocline intersects the seafloor) at the edge of the brine pool (right). Note floating garbage in the brine pool.

E. Mediterranean DHABs such as Discovery Basin (Figure 4) are found more than 3,000 m below sea level. There is very little mixing of these brines with the overlying seawater due to their high density (typically ranging from 1.13 to 1.35 × 10^3^ kg m^−3^ relative to Mediterranean seawater 1.03 × 10^3^ kg m^−3^). The steep halocline that results, can be only a few meters or less in thickness, oxygen concentrations drop to undetectable at the base of the halocline, and salinity increases dramatically, often to around 10 times normal seawater [56]. The chemical compositions of the different basins are distinct, with widely varying concentrations of sulfide, methane, and various cations and anions ([56] Supplementary Material) (Table 1). Some brines, such as that found in Discovery Basin, are athallasohaline, with Mg^2+^ concentrations up to 5,000 mM compared with 300–650 mM in other basins, and ca. 60 mM in regular seawater. All other basins reported here are thalassohaline. Sodium concentrations can vary widely in the different basins. For example, sodium is 70 mM in Discovery Basin brine, and almost 4,700 mM in Atalante Basin brine [56]. Combined with the very high pressures associated with the depths of these basins, DHABs represent some of the most extreme habitats on Earth.

In the water column, abundant chemosynthetic bacterial communities along DHAB oxyclines (e.g., [56,57,58,59,60]) appear to support active pelagic protist communities there [25,33,42,43]. Moderately hypersaline systems are known to sustain rich and diverse communities of mostly halotolerant eukaryotes [13]. Habitats with salinities in excess of 30% are not thought to harbor significant protist diversity [14,15,16,17,18] (but see opposing viewpoint in [19]). Although previous studies suggested habitats with salinities in excess of 30% did not harbor significant protist diversity (see above), initial investigations into protist diversity in several Eastern Mediterranean DHAB haloclines and brines using DNA-based [42] and RNA-based [25] molecular approaches suggested that these pelagic habitats not only harbor diverse protistan communities, but that these communities are largely unique to the water columns of these basins and share little overlap with overlying waters with typical marine salinity and oxygen tension.

The first indication that haloclines and brines of DHABs support active microbial eukaryotes came from two studies in 2009 that presented profiles of SSU rRNA genes in clone libraries, one based on RNA extracted from halocline samples from Atalante basin (upper halocline 39 PSU, lower halocline 365 PSU) [25] and one based on DNA extracted from haloclines and brines of Bannock (halocline 246 PSU and brine 280 PSU) and Discovery basins (halocline 70 PSU) [42]. Similar to Hamelin Pool microbialite samples, abundant signatures of alveolates were recovered from Discovery and Bannock samples (75% of OTUs at 98% sequence similarity), most of which were from dinoflagellates (62%) and ciliates (12%) [42]. Fungi were the third most abundant group (17% of OTUs), particularly in brine samples of both basins. Signatures were also recovered from stramenopiles, euglenozoans, position="float", plants, cercozoans and kinetoplastids. Due to the steep density gradient typical of haloclines, organic material (dead cells and detritus) accumulates at the top of haloclines and likely is partly responsible for fueling the active chemosynthetic prokaryotic communities on which these protists and fungi feed. Phagotrophic protists are known to be quite successful along oxyclines where prokaryotes are abundant [61,62,63] and so it follows that they are also successful within these haloclines. Fungi are active remineralizers of organic material that accumulates at these haloclines, and some which sinks into the brine. The presence of plant and metazoan signatures within the DNA-based clone libraries reflects their likely detrital origin. Nonetheless, the dominant protist signatures from Bannock and Discovery Basin haloclines and brines [42] are distinct from the picture of open-ocean pelagic communities in the photic zone that are usually dominated by stramenopiles and pigmented picoplankton taxa [64,65,66].

The density of protists that halocline and brine habitats can support is significant. For example, in a study of Thetis basin, which has one of the highest salt concentrations reported for DHABs (340 PSU), protist counts of ca. 0.6 × 10^4^ per liter were reported in the anoxic brine [43]. The study of Thetis was based on RNA extracted from water samples, which is less likely than DNA to originate from dead or inactive cells, and identified fungi as the most diverse taxonomic group of eukaryotes in the brine (38% of OTUs based on 98% sequence similarity), followed by ciliates and stramenopiles, each accounting for 20% of phylotypes. Ciliate OTUs detected in the Thetis study were closely related to sequences detected in surveys of other DHABs, suggesting specific adaptations of ciliates to these habitats [43]. In addition to OTUs from dinoflagellates, haptophytes, choanoflagellates and jakobids, OTUs affiliating with marine stramenopiles (MAST) were detected in the brine samples, expanding the known salinity range of these taxa. Beta-diversity analyses supported the uniqueness of brine *vs*. halocline communities [43].

The RNA-based study of SSU rRNA gene signatures from Atalante basin halocline by Alexander *et al*. [25] also supported the presence of active protists in these hypersaline habitats. Almost the same number of OTUs (99% sequence similarity) were recovered from the upper halocline as from the extremely hypersaline lower halocline (43 and 42, respectively). In that study, alveolates also dominated the protist community, and ciliates were the most common group of alveolates in both the upper and lower halocline (18 and 21 OTUs, respectively). Only 12 OTUs (including seven ciliate, two choanoflagellate, and one each fungal, radiolarian, and jakobid OTUs) were shared between the two samples that were only separated by ~1.5 m water depth but differed in salinity by 324 PSU. Although salinity differences may be the primary driver behind observed differences in protist community composition, other environmental factors, including oxygen and ammonia concentrations likely also play a role [25]. Fungal and radiolarian OTUs were common in the upper halocline (39 PSU), but only a single representative of each group was detected in the lower halocline (365 PSU). Stramenopile, haptophyte, rhizarian and chlorophyte signatures were detected exclusively in the upper halocline, while cryptophyte and diverse dinoflagellate OTUs were detected exclusively in the lower halocline [25]. Comparisons of protist communities found in Bannock, Discovery, and Atalante using Jaccard indices support the notion that unique basin chemistries select for unique protist communities (e.g., [42] comparison of Bannock and Discovery). This is supported by a recent broad comparison of eukaryotic communities in many different DHAB haloclines and brines using T-RFLP by Filker *et al*. [55].

**Figure 5:**
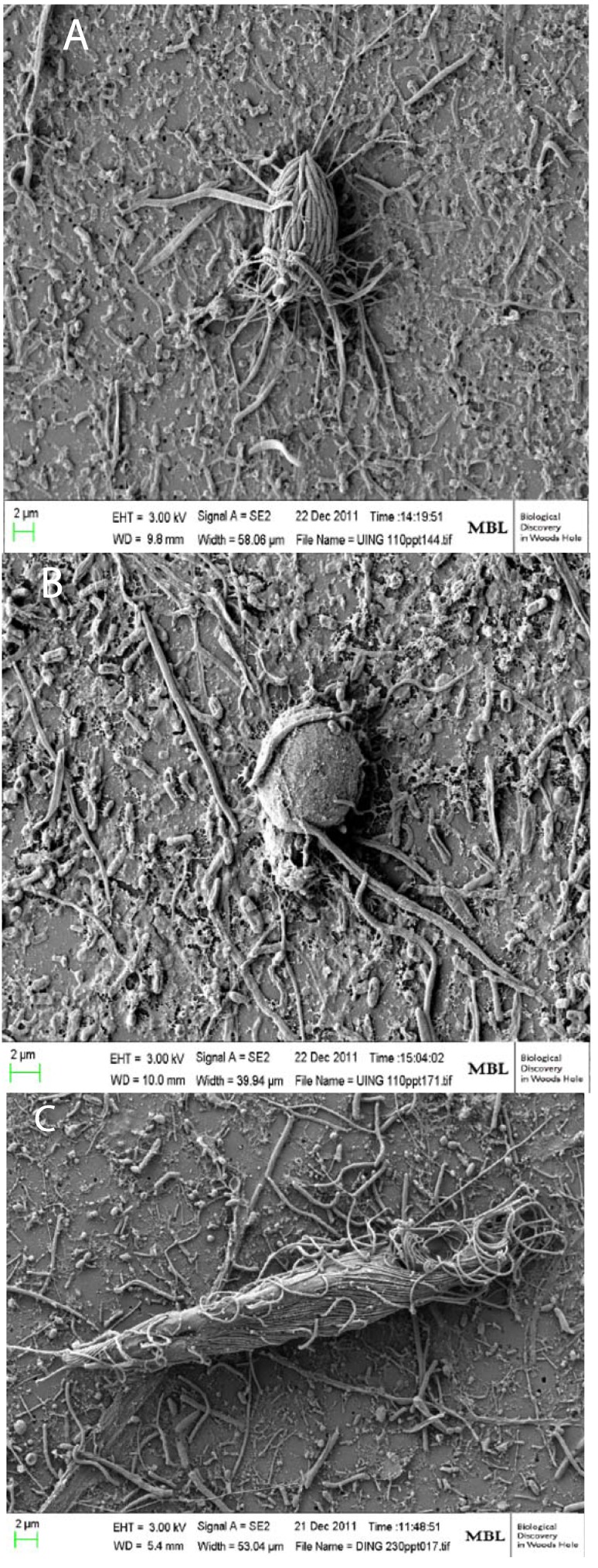
Scanning electron micrographs of microbial eukaryotes from the water-column haloclines of Urania and Discovery Basins in the Eastern Mediterraean Sea. (**A**) A scuticociliate morphotype consistently associated with epibiotic bacteria (**B**) that has been found to be the most abundant eukaryotic morphotype in the Urania halocline [45]; (**B**) A flagellate in Urania halocline. (**C**) A larger ciliate associated with long (10–20 µm), thin, filamentous bacteria, which was the most abundant eukaryotic morphotype in the Discovery halocline [45]. Scale bars = 2 µm. Photos by William Orsi.

Confirmation of active/living protists in different DHAB samples was obtained using scanning electron microscopy (SEM) and fluorescence *in situ* hybridization (FISH) to visualize intact cells on filters. SEM images of intact ciliates and flagellates (Figure 5) showed that most ciliates on halocline and brine filters hosted prokaryotic epibionts. The role of these symbioses in DHAB habitats is under investigation. In the course of examining SEM filters from Discovery Basin halocline water samples, it was noted that abundant kinetoplastid cells were present. Kinetoplastids have been reported previously from anoxic and high-salt environments [13]. Kinetoplastid-specific PCR primers were used to amplify kinetoplastid signatures from brine and halocline water samples from six basins with differing chemistries [33]. Amplifications were successful only for halocline samples from three of six basins (and not for normal seawater controls). FISH probes were developed using the SSU rRNA gene sequence of an “unidentified clade” of kinetoplastid signatures that was observed to dominate the Discovery Basin halocline clone library. It was revealed using FISH that this clade represented up to 10% of the total protist community in the Discovery Basin halocline (6.4 × 10^3^ kinetoplastids per liter belonging to this clade). This clade most likely represents a new genus [33]. Finding signatures of this “unidentified clade” of kinetoplastids from only halocline filters from three of six basins lends further support to the notion that unique basin chemistries are driving protist community composition.

## 3. Commonalities between Protist Communities in DHABs and Hypersaline Shallow Water Mat Communities (Hamelin Pool and Guerrero Negro)

Direct comparisons between protist communities detected in DHAB halocline and brine waters with those in shallow water hypersaline sedimentary microbial mat communities is difficult for a number of reasons. First, communities in benthic and aquatic habitats are normally quite distinct, and second, the sampling and methodological approaches used in each of the studies discussed here are different. Nonetheless there are groups of protists and fungi that are common to both shallow hypersaline mat and DHAB environments, including diverse alveolates, stramenopiles, and fungi, as well as radiolaria. Within alveolates, groups that were common included ciliates (including signatures of Oligohymenophorea, Spirotrichea, Heterotrichea, Plagiopylea, and Phyllopharyngea) and uncultured Group I and Group II alveolates, as well as diverse dinoflagellates (including signatures of Gonyaulacales, Prorocentrales, Peridiniales, Gymnodiniales, Syndiniales). Stramenopile signatures shared include those from Labyrinthulidae, Bicosoecida, and Marine Stramenopile Group 3 (MAST-3). Radiolarian sequences shared between DHABs and Hamelin Pool microbialite samples included signatures of Polycystinea and Acantharia. In both Hamelin Pool microbialites and DHAB haloclines and brines many sequences of novel eukaryotes with no close homologues in public databases were recovered, suggesting these habitats host unusual protist and fungal communities. The taxonomic identification of the eukaryotic cells behind those signatures, as well as the determination of the environmental role of those eukayotes and their impact on carbon and other nutrient cycling in hypersaline habitats, is a fascinating avenue for future investigation. Furthermore, groups of protists are identified here that are common to different hypersaline habitats. By studying cultured representatives of these groups we will gain a better understanding of the physiological underpinnings behind their ability to adapt to hypersaline conditions. Future studies of heterotrophic grazing impacts in hypersaline water columns and sediments will enhance our understanding of carbon and other nutrient cycling in these habitats.
